# Intellectual and Developmental Disabilities: Denmark, Normalization, and De-Institutionalization

**DOI:** 10.3389/fpubh.2014.00161

**Published:** 2014-09-24

**Authors:** Joav Merrick, Peter Uldall, Jakob Volther

**Affiliations:** ^1^Health Services, Division for Intellectual and Developmental Disabilities, Ministry of Social Affairs, Jerusalem, Israel; ^2^Department of Paediatrics and Adolescent Medicine, Rigshospitalet, Copenhagen, Denmark; ^3^Residential Care Kellersvej, Søborg, Denmark

**Keywords:** intellectual and developmental disabilities, Denmark, normalization, de-institutionalization, epidemiology

## Introduction

The Danish physician Jens Rasmussen Hübertz (1794–1855) conducted one of the first epidemiological studies on insanity, intellectual and developmental disabilities (IDD) published in 1843 (1) and found a total number of persons with IDD of 2,000 and a prevalence of 0.9 per 1,000 population (2). After graduation, he had worked during a dysenteriae epidemic and this experience together with a trip to Germany in 1841 awakened his interest in both mental illness and IDD. His observations in Germany and also in his travels in Denmark conducting his research made him a spokesman for reform in the care and service for people with mental illness and IDD in Denmark. His research also resulted in his differentiation between mental illness and IDD and gradually he placed all his attention to the establishment of a service for people with IDD in Denmark.

In 1852, he traveled to Switzerland to meet the physician Johann Guggenbühl (1816–1863), who in 1839 had established the first known residential care facility for children with IDD in Abendberg, a mountain near Interlaken. Guggenbühl believed in the fresh air from the mountains as part of his treatment together with good nutrition, baths, massage, exercise, and also to some extent, medication. This trip inspired Hübertz to come back and raise private funds for the establishment of the first institution in Denmark for 20 children with IDD in November 1855. With his work, he therefore became the father of the modern service for people with IDD in Denmark, but died of stroke 2 weeks after the first children moved into Gamle Bakkehuset (the Hill House).

Gamle Bakkehuset has a very interesting story, which is typical for many of these institutions around the world (3). It was built back in the 1520s at the outskirts of Copenhagen, today a museum. It has served a number of functions over the years as a farmhouse, inn, private home, official home, psychiatric hospital, residential care for children with IDD, and now a museum and the first floor guest apartment for 2 h for dignitaries. It is particularly associated with the Danish Golden Age in the 1800s, when it was a popular summer social venue for the literary and intellectual establishment with, for example, the author Hans Christian Andersen (1805–1875) visiting one of the greatest Danish actresses Johanne Luise Heiberg (1812–1890) there.

In 1865, the second residential care center was established (Keller) in Copenhagen and by 1940 Denmark would have all over the country 10 such centers with a total population of around 5,000 persons with IDD (4).

## History

These institutions and the work with this population was initially done by private persons or non-for-profit organizations, supervised by the Ministry of Religious Affairs and Education, but the responsibility in 1959 transferred to the State and the Ministry of Social Affairs.

In Denmark, the National Assistance Act of 1933 declared that institutional care, treatment, education, and foster-home care should be the responsibility of the government (5). This social reform defined the responsibilities for society and the service toward the weak and it was again reaffirmed by the passage of the National Assistance Act in 1961, and supplemented by special acts on the deaf, the blind, and the “mentally retarded” (5).

Since 1937, several laws have been introduced in order to prevent disease and mortality in infants during their first year of life and in 1945, the Maternity Welfare Act provided free routine examinations of all expectant mothers by a physician and a midwife. A unique nurse health visitor program was established in 1939 in order to provide home visits to check expectant mothers and afterward regularly follow the health of the children in their homes. In 1946, another law was passed to provide for nine free preventive medical examinations of infants from birth to their seventh year (5) and afterward the health control is taken over by the school physician and nurse in a regular examination program. In case of development problems, the family physician (the gatekeeper in primary health in Denmark) is contacted and if required referred to the regional pediatric hospital ambulatory services (5). The preventive health work is carried out by both the health visitor and the general practitioner with the service provided free through the National Health Service.

In 1959 by Act 192 of June 05 (Mental Retardation Act) Denmark established a state obligation for the care and service for people with IDD and the Mental Retardation Service was established under the jurisdiction of the Ministry of Social Affairs (5). The service system divided Denmark into 12 regions, each region administered from 1 of the 10 residential care centers. However, a single superregional training program for care personnel was operated in Copenhagen and from 1966 a Children’s Hospital (Vangede). The region of Copenhagen was different with two teams, one for children service and one for adult services.

Then Mental Retardation Service was administered by a board of directors working in close collaboration with the regional centers (5). The eight member board of directors was appointed by the Minister of Social Affairs for a 4-year period with one member as a representative of the National Health Service, one from the Ministry of Education, and one representative from the national association of parents. The other five members were persons or professionals with knowledge and interest in this field. The board was chaired by the Director of the Service, who was appointed by the King (5).

The board of directors had the responsibility to carry out the act, to monitor development within the field, and to present the Minister of Social Affairs with new ideas, development, and new programs.

At the regional level, a board of directors was appointed in order to monitor the function of the center(s) and develop further services and projects within the region. Each regional center was administered by an administrator, a chief physician, a director of social work, and a director of education (5). The center team was responsible to the (national) board of directors for all activities within its region and the board also had a representative from the parent association (5).

In 1967, the Kennedy Center was established to conduct research on a national level in rare genetic diseases causing IDD and visual handicap. In 2012, this center was merged with the Clinic for Genetic Diseases at Rigshospitalet (the central hospital for Denmark in Copenhagen) and provides today health service via national centers for Fragile X, PKU, Rett syndrome, Alkaptonuria, Vision Impairment, IDD, and more.

## Development

The period 1945–1959 was characterized by the medical model with the idea that this population could be treated or educated in large residential care centers under medical supervision. This medical model resulted in lobotomy, sterilization, and also castration (6). Toward the end of the period, public criticism of the conditions in the institutions resulted in new laws.

The 1960–1967 was characterized by shift in concept toward normalization, equality, and human rights. During this period, professionals such as educators, social workers, psychologists, and others gained more influence in mental health care and psychiatry resulting, in which teaching, psychotherapy, and psychological testing came to play a greater role. However, much of the treatment was still administered in large institutions, and lobotomy, sterilization, and castration were still used, albeit less often than in the past (6). At the close of this period, a greater focus on the rights of this population was seen with amendments to laws and abolishment of sterilization with the mental health care law in 1967 and a ban on all forms of corporal punishment (1968) and the elimination of censorship of letters (6).

The period from 1968 to 1980 was characterized by further discussions on psychiatry and mental health issues, ideas about self-determination and democracy, normalization, group homes, and care in the local community. This led to closure or re-organizing larger institutions into smaller units and placement in the local community. This period saw many changes with a new Social Assistance Act (1976), psychiatry services transferred from state to local county responsibility (1976), and the transition of mental health care and care for people with IDD to the county level (1980) (6). Lobotomy, sterilization, and castraction became totally abandoned.

## Father of De-Institutionalization or Normalization

Niels Erik Bank-Mikkelsen (1919–1990) introduced the concept of normalization in the 1959 Mental Retardation Act. Normalization was seen as a way to ensure that this population received the same legal and human rights given to other citizens (5, 7, 8). Together with the Bengt Nirje (1924–2006) from Sweden, they were instrumental in changing the paradigm toward normalization in many countries around the world.

In 1968, Bank-Mikkelsen received the Kennedy Foundation Award in recognition of his work, which culminated with the 1980 Law and the abolishment of the Service for the Mentally Retarded at the Ministry of Social Affairs, since all service and care from then had to be provided by the counties and no longer the State.

## Denmark Today

The transfer from a central government control to a regional county control has its pros and cons. One disadvantage is the lack of a central data register on the population with IDD in Denmark, which, for example, has resulted in less research output from Denmark in this field since the 1980s.

On the practical level, the large institutions have been replaced by smaller unit of protected apartments or group homes with trained staff, some places with health staff, while others are using the local general practitioner and the National Health Service facilities. Overall, the intention of normalization was to create living facilities with privacy and home environment and integration in society as much as possible and utilization of services like the general population.

A recent research project (4) looked at the health status of adults with IDD in Denmark. This study was somewhat complicated, because of the loss of a central register for this population, but by a combination of six registers (like the Central Patient Register, Mortality Register, Psychiatric Central Register, and the Central Personal Register), it was possible to identify a cohort of 73,445 persons with IDD from 1968 to 2014 with 50,455 still alive.

The study (4) revealed a prevalence of IDD in Denmark at age 15 years of 15–18 per 1,000 and a prevalence of moderate, severe, and profound IDD of 3.5 per 1,000. The study also found a significant increase of mortality (cardiovascular, cancer, and suicide/accidents-related diseases) for both genders by a factor 6 in the 20–40 years age group decreasing to a factor 2 in the age group of 70–80-year olds. Or in other words, an adult with IDD will on average live 14.5 years less than the general Danish population. The overall aim of the report was to investigate, find documentation, and discover possibilities to reduce inequality in health for people with IDD (4).

## Conclusion

Denmark has been one of the leaders in the normalization movement, which has resulted in the 10 larger residential care centers closed or decreased to smaller residential unit. New group homes are smaller with a professional staff, but health care is mainly provided by general practitioners, just like the general population. The normalization process also resulted in administration transferred from a central to a regional responsibility, which has resulted in a lack of overview and surveillance of this population and there have been very few studies that have documented the effect of the normalization process on health.

We are sure that the genetic revolution will have an impact on the future of this population, which can already be seen in the fewer numbers of persons with DS being born today in Denmark (9). Further development of technology and prenatal screening will have an impact on the future size of the population of people with IDD in Denmark. In view of the recent study on the health status of this population (4), the Danish health authorities, service, and government need to take a closer look at the health care and service provided in order to improve the health service, care, and surveillance of this specific population.

## Conflict of Interest Statement

The authors declare that the research was conducted in the absence of any commercial or financial relationships that could be construed as a potential conflict of interest.

## References

[1] Hübertz JR Om Daarevæsenets Indretning I Danmark. Copenhagen: Andreas Frederik Høst (1843).

[2] Dupont A 140 Years od Danish studies on the prevalence of mental retardation. Acta Psychiatr Scand (1989) 79(Suppl 348):105–12 10.1111/j.1600-0447.1989.tb05220.x 2801173

[3] Merrick J Hjorth PS Gården L, editors. Childhood. A Book on Child Health and Development During the Past 300 Years. Copenhagen: Danish Nursing Association (1988).

[4] Flachs EM Michelsen SI Uldall P Juel K Sundhedstilstanden Iblandt Voksne Med Udviklingshæmning. Copenhagen: Statens Institute Folkesundhed (2014).

[5] Bank-Mikkelsen NE A Metropolitan Area in Denmark: Copenhagen. Washington, DC: President’s Committee on Mental Retardation (1969).

[6] Svendborg Museum. Available from: http://www.anbragtihistorien.dk/english/periods.html

[7] Kugel RB Wolfensberger W, editors. Changing Patterns in Residential Services for the Mentally Retarded. Washington, DC: President’s Committee on Mental Retardation (1969).

[8] Ericsson K The principle of normalization: history and experiences in Scandinavian countries. Presentation, ILSMH Congress Hamburg (1985).

[9] Ekelund CK Jørgensen FS Petersen OB Sundberg K Tabor A Danish Fetal Medicine Research Group. Impact of a new national screening policy for Down’s syndrome in Denmark: population based cohort study. BMJ (2008) 337:a2547 10.1136/bmj.a2547 19039015 PMC2590884

